# The hazards of perception: evaluating a change blindness demonstration within a real-world driver education course

**DOI:** 10.1186/s41235-019-0165-4

**Published:** 2019-05-21

**Authors:** Daniel O. A. Gunnell, Melina A. Kunar, Danielle G. Norman, Derrick G. Watson

**Affiliations:** Department of Psychology, The University of Warwick, Coventry, CV4 7AL UK

**Keywords:** Driving behavior, Overconfidence, Change blindness, Education, Behavioral change

## Abstract

Overconfidence in one’s driving ability can lead to risky decision-making and may therefore increase the accident risk. When educating people about the risks of their driving behavior, it is all too easy for individuals to assume that the message is not meant for them and so can be ignored. In this study we developed and assessed the effect of a road safety demonstration based around the phenomenon of change blindness within a real-world Driver Awareness Course. We collected quantitative and qualitative data to evaluate the effectiveness of the demonstration in both a police-led environment (Experiment 1) and a laboratory environment (Experiment 2). We also compared the change blindness intervention to two control tasks. The results showed that participants’ self-reported ability to spot important visual changes was reduced after the change blindness demonstration in both experiments, but was not reduced after participation in the control tasks of Experiment 2. Furthermore, participants described the change blindness demonstrations positively and would recommend that they were shown more widely.

**Electronic supplementary material:**

The online version of this article (10.1186/s41235-019-0165-4) contains supplementary material, which is available to authorized users.

## Significance

As well as offering valuable academic insights, research into visual perception and attention is applicable to many real-world tasks. In the current study, we designed, implemented and evaluated a novel driver education intervention based on attention theory. The research was performed in collaboration with Dorset Police (UK) Driver Education Unit, who offer courses to road users who commit a driving offense such as speeding, talking on their mobile phone or driving through a red light. Working with our collaborators, we chose to target the key driving behavior of overconfidence in one’s observational abilities whilst driving. We designed the intervention to be easily incorporated into the course and measured participants’ views on the intervention using a mixed-methods approach. Combining qualitative data with quantitative methods added a depth to our research that would not have been possible using a single-method approach. The work has been presented to key academic and emergency service personnel and a representative of the UK National Driver Awareness Course.

## Introduction

Flaws and limitations in visual processing are well known within the psychological literature and yet, because everyday perception *seems* so natural and complete to us, the layperson can be unaware of such weaknesses. People have the impression that their vision is seamless, continuous and unlimited, and because of this they may overestimate their ability to perceive the world around them whilst performing everyday tasks. With relevance to the topic of driving behavior, failing to notice or see objects can have catastrophic consequences for the driver and for other road users. Thus, making people aware of their attentional limits might encourage them to consider such limitations whilst driving, leading to safer driving behavior. For example, being more aware of the limits of vision and attention might make people look just a little bit longer and more carefully before pulling out of a junction, to make sure that they have not missed something.

This general approach is not without precedence; demonstrations of visual flaws have been used successfully in other domains to improve safety. For example, a phenomenon named motion-induced blindness (Bonneh, Cooperman, & Sagi, 2001) has reportedly been used to demonstrate to aircraft pilots the importance of moving their head and eyes around when scanning the environment, preventing the pilot from focusing on one particular spot which can cause a failure to notice stationary objects. Other work has shown that people miss a large proportion of targets if they are rare or the display is complex, which has implications for real-world search tasks such as airport security/baggage screening or searching for anomalies in medical images (e.g., Kunar, Rich, & Wolfe, 2010; Kunar & Watson, 2011, 2014; Kunar, Watson, Taylor-Phillips, & Wolska, 2017; Russell & Kunar, 2012; Van Wert, Horowitz, & Wolfe, 2009; Wolfe, Horowitz, & Kenner, 2005; Wolfe et al., 2007). Accordingly, with the view that laboratory studies are useful tools to help in real-world tasks, in the present work we developed a demonstration of change blindness to be used in driver education. This demonstration was evaluated within a real-world UK driver education course using both quantitative and qualitative methods.

### Change blindness

Change blindness (Rensink, O’Regan, & Clark, 1997) is the name given to the finding that it is difficult to notice changes that occur in a scene if those changes occur whilst one’s vision is temporarily disrupted—for example, during an eye blink (O’Regan, Deubel, Clark, & Rensink, 2000) or even an eye movement (Grimes, 1996; Henderson & Hollingworth, 1999). Examples of the consequences of change blindness can be found in many real-world situations. For example, in one study an experimenter engaged the attention of a pedestrian and began a conversation. Halfway through the conversation, workmen walked between the participant and the experimenter, blocking the participant’s view with a door, while a confederate swapped places with the original experimenter (Simons & Levin, 1998). In this study only 50% of participants noticed the switch.

### Demonstrating change blindness

The change blindness phenomenon can be demonstrated relatively easily by repeatedly presenting two pictures, let us call them picture A and picture B, one after the other. Picture B is the same as picture A except that a single change has been made to it. For example, in a driving scene, picture B might be the same as picture A except that a car or pedestrian has been removed from the image. In a typical change blindness demonstration, picture A will be presented followed by a blank screen and then picture B followed by a blank screen, and so on (e.g., see Rensink et al., 1997). The interleaved blank screens simulate and have the same effect as making an eye blink (or eye movement) by masking the transients between the two images that would normally indicate the location of a change. This picture A–blank–picture B–blank sequence repeats and the task is to try to find the difference between the two pictures. Typical findings show that people are exceptionally bad at spotting the difference between the two images even with prolonged viewing times (Rensink et al., 1997).

Numerous factors influence how difficult a change is to observe in a change blindness task. For example, certain changes are easier to see than others. If a change is made to an image that is semantically inconsistent with the original image it is detected more readily (Stirk & Underwood, 2007). Furthermore, not all change blindness paradigms require visual occlusion; change blindness can still occur when something changes gradually in a scene or when transients are introduced (Simons, Franconeri, & Reimer, 2000; Watson & Kunar, 2010). When changes occur in this way, older adults find it more difficult to detect these changes than younger adults (Batchelder, Rizzo, Vanderleest, & Vecera, 2003). In addition, cognitive load, bought about by trying to complete two tasks simultaneously, can also modulate change detection performance. For example, when participants hold a naturalistic conversation while viewing change blindness images, they are less likely to observe the changes in the images (McCarley et al., 2004). McCarley et al.’s study also showed that the context of a change is important; meaningful changes to a scene were detected more quickly than less-meaningful changes. Finally, cultural variation in change detection performance has also been reported (Masuda & Nisbett, 2006).

The facts regarding how change blindness occurs and under what circumstances changes are harder to detect are potentially important for driver safety. It is possible that change blindness plays a factor in road safety. Given that change blindness can occur under single-task laboratory conditions (Rensink et al., 1997) and that simple tasks such as holding a conversation can exacerbate the change blindness effect (McCarley et al., 2004), it stands to reason that a complex task such as driving may also make change detection more difficult. This might be especially the case given that a driver must continually update their representation of the world as they move through it at speed. We investigate whether change blindness demonstrations can play an important role in driver education in terms of highlighting that people may not see as much as they think they do.

### Driver capability and overconfidence

Overconfidence in one’s ability has been found in a variety of domains from academic study (Clayson, 2005) and financial decision-making (Statman, Thorley, & Vorkink, 2006) to driving ability (Svenson, 1981; for a review, see Moore & Healy, 2008). Indeed, in one study 70–90% of drivers reported that their driving was better and less risky than that of the average driver (Svenson, 1981). Similarly, participants usually overestimate their ability to detect changes in a visual scene. When participants were asked whether they would see certain changes within a scene, they stated confidently that they would. However, when other participants were tested via a change blindness technique, the actual detection rate for those changes was low (Levin, Momen, Drivdahl IV, & Simons, 2000). Although it does not appear possible to teach participants to see changes more efficiently (Rensink et al., 1997), it is possible that by increasing awareness of visual limitations, people will be more vigilant overall and perhaps more likely to avoid engaging in distracting tasks.

Overconfidence can be dangerous and can lead to risky decision-making and potentially hazardous consequences. Sandroni and Squintani (2004) conducted a literature review focusing on health, driving risk and overconfidence. They concluded that overconfidence in one’s driving may result in poor decisions such as not purchasing adequate insurance. The review also presents evidence suggesting that traditional driver education and training designed to educate drivers about the risks and hazards of driving may in fact be exacerbating the overconfidence problem by increasing participants’ confidence in their own abilities. For example, Katila, Keskinen, and Hatakka (1996) conducted an evaluation of special courses run in several countries designed to make drivers safer on slippery roads. The authors suggest that one of the reasons why these courses are not effective is that they may be making drivers feel more capable and are increasing drivers’ confidence in their ability to handle loss-of-control situations. This is because the courses promote practicing routines in controlled situations but these routines may not transfer well to real-life scenarios. One consequence of this is that drivers who have greater confidence in their own abilities may feel comfortable driving more dangerously. However, more recent work from Katila, Keskinen, Hatakka, and Laapotti (2004) suggests that this may be an oversimplification of the link between overconfidence and accidents, and that higher confidence alone may not predict safety but rather one’s skills, and how those skills are used is of critical importance.

Such undesirable effects are not without precedence; it has been shown that adding safety measures to vehicles is not always effective at reducing accidents on the road. For example, Peterson, Hoffer, and Millner (1995) examined data on the effects of introducing airbags into cars. They concluded that drivers compensated for the addition of airbags by adopting a more aggressive driving style, which negated the benefit for the driver and increased the risk to other road users. There are many explanations for these kinds of effects where the addition of a safety measure is not met with the expected increase in driver safety; for example, Hedlund’s compensation index (Hedlund, 2000) and the controversial theory of risk homeostasis (Wilde, 1982). The general consensus of these theories is that in many cases the introduction of a safety feature or procedure results in the perception that the individual is now safer and so can offset this perceived increase in safety by taking greater risks (Vrolix, 2006).

There are a variety of models which can be used to try to understand and then change behavior. One such model is the theory of planned behavior, which focuses on participants’ attitudes, subjective norms and their perceived behavioral control (Ajzen, 1991). Another model of behavioral change, the Capability Opportunity Motivation-Behavior (COM-B) model, highlights the importance of *capability*, *opportunity* and *motivation* for influencing behavior (Michie, van Stralen, & West, 2011). Of particular relevance to the current study is capability, which is defined as “the individual’s psychological and physical capacity to engage in the activity concerned. It includes the participant having the necessary knowledge and skills” (Michie et al., 2011, p. 4). Behavioral change interventions, therefore, can target a particular level, or multiple levels, of the COM-B framework in order to affect a target behavior. When behavioral change interventions focus on capability, it is usually the case that they focus on improving capability or giving participants the capacity and the means to perform a behavior, such as encouraging healthy eating (Atkins & Michie, 2013) and medical adherence (Jackson, Eliasson, Barber, & Weinman, 2014). However, the capability component of the COM-B model could theoretically be applied to reduce an individual’s confidence in their own knowledge or skill in performing an action in order to modify their behavior.

### Demographic differences in overconfidence

Overconfidence, in certain contexts, has been found to be related to both age and gender. Men have been found to be generally more overconfident than women in a variety of different domains, such as confidence in their academic test answers (Bengtsson, Persson, & Willenhag, 2005), particularly when the answers are incorrect (Lundeberg, Fox, & Punćcohaŕ, 1994), confidence when making investment decisions (Barber & Odean, 2001) and confidence in competitive tasks (Niederle & Vesterlund, 2007). However, in the field of driving behavior the data are not so clear. Young male drivers are more likely to underestimate their chance of an accident (Finn & Bragg, 1986) and perceive driving as less risky (Rosenbloom, Shahar, Elharar, & Danino, 2008) relative to their peers and older drivers. In contrast, research which has focused on assessing driver capability and comparing this to self-report ratings of confidence of driving ability has shown that male drivers may not be any more overconfident in their abilities than female drivers (Mynttinen et al., 2009). The current work also touches on another variable that may interact with overconfidence, a person’s age.

Studies examining the relationship between age and overconfidence have shown seemingly conflicting results. Menkhoff, Schmeling, and Schmidt (2013) found in an investment context that older participants were more overconfident, whereas, in a different domain, Pliske and Mutter (1996) found that older adults were more accurate in their judgments of their own performance on a general knowledge test. In addition, as previously discussed, younger drivers are more likely to be overconfident than older drivers (Finn & Bragg, 1986; Rosenbloom et al., 2008). Given this set of conflicting findings, we also assessed the effects of age and gender in the present work.

### A method to reduce overconfidence

Previous work, although not explicitly COM-B based, has shown that it is possible to reduce participants’ overconfidence in their abilities. For example, although people are generally overconfident in their answers to general knowledge questions, it is possible to reduce such overconfidence (Arkes, Christensen, Lai, & Blumer, 1987). One way to achieve this is to present questions which appear to be easy but are in fact challenging and then provide the participants with feedback on their answers. Arkes et al. (1987) applied this method and showed that individuals were not as confident in their answers to subsequent general knowledge questions once they had been made aware of their performance on questions they had thought were easy. Pulford and Colman (1997) showed that it is not the feedback which is important in this case, but rather the mismatch between perceived and actual difficulty. In the current study, we adapt this approach for use in driver education by demonstrating to drivers how seemingly easy-to-spot changes, in a visual scene, may go unnoticed.

### Overview of the current work

In the current study we evaluated the feasibility and effects of introducing a driving-related, change blindness task into a real-world driver education course. Of particular interest was the effect that the change blindness demonstrations might have on participants’ self-reported observational abilities. In Experiment 1, participants in a police-led driver course first completed a pre-test questionnaire to obtain their baseline views of how observationally skilled they were. A series of change blindness demonstrations were then shown, followed by a post-test questionnaire that re-assessed participants’ views. The questionnaires were designed to provide both quantitative and qualitative data. This mixed-methods approach allowed for a quantitative analysis of participants’ confidence regarding their observational and other abilities, while the qualitative data added detail and complementary information. In Experiment 2, we replicated the experiment in the laboratory, so that any perceived influence of a police representative being the experimenter was eliminated. In this experiment we also compared responses for participants who independently completed either the pre-test or post-test questionnaire, so that participants’ responses were not affected by “test–retest” factors or expectancies of completing the same questionnaire twice. Finally, we examined the effect of other “pseudo-interventions” on people’s self-reported observations to examine whether the change blindness aspect of the demonstration was particularly effective or whether any demonstration related to driving or self-confidence could elicit the same effects. For this we used: a driving-themed visual search task (using identical stimuli to Experiment 1); and a multiple-choice question task designed to induce a “sense of failure” and undermine self-confidence (see also Arkes et al., 1987).

## Experiment 1

### Method

#### Participants

A total of 160 participants (61 female, 96 male, 3 declined to answer) (age 18–85 years, mean = 44.1 years, standard deviation (SD) = 14.7 years) attending police Driver Awareness Courses (Dorset Police, UK) took part in the demonstration. Participants voluntarily attended the course after they had been caught committing a minor traffic violation, as an alternative to receiving points on their driving license. In respect to the demonstration, participants were informed that their participation was completely voluntary and that any answers they gave would be treated anonymously. Participants had the right to refuse to answer any parts of the questionnaire should they wish to do so. They were also specifically told that their answers to the questionnaires would have no bearing on their successful completion of the course. Participants were tested in groups of approximately 20. Full ethical approval for this work was granted by the Department of Psychology Ethics Board of the University of Warwick.

#### Materials and stimuli

Six change blindness examples were created. When designing the examples, several considerations were taken into account: it was important that the images related to driving scenes to match the content of the course (see Lees, Sparks, Lee, & Rizzo, 2007); it was necessary to minimize any potential cues that might lead to an artificial improvement in change detection across repeated demonstrations (e.g., ensuring that the change was not always in the same location and was not always the same type of object); and changes that might be particularly easy to detect (e.g., changing many parts of the scene, changing whole regions or introducing semantic inconsistences; Stirk & Underwood, 2007) were avoided to ensure an adequate demonstration of the change blindness phenomenon.

**Fig. 1:**
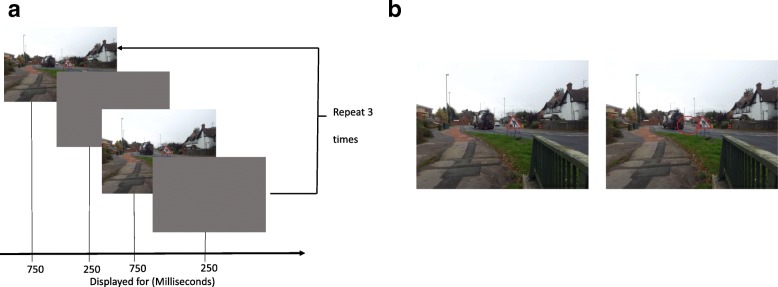
Example of the procedure for a single change blindness demonstration. **a** Participants viewed the interleaved images for a total duration of 6 s. **b** Following this, the instructor clicked on a “display the change” button which presented the two images side by side, with the change highlighted by an outline circle

The images were of typical driving situations and depicted varying traffic conditions and environments. In total, 12 images were created; we then piloted these images and asked participants to rate how difficult the change was to observe and the relevance of the change to a driving scenario. The final six images were chosen to balance both difficulty to observe the change and driving relevance. For all images, identifying features such as number plates and road names were blurred out. Part of one of the images from each image pair was modified in order to create a difference between the two images. During a change blindness example, the two associated image pairs were presented sequentially with a blank gray screen interleaved between them. Each image was presented for 750 ms and the intervening blank screen for 250 ms. The total duration of each demonstration was 6 s (see Fig. 1). Side-by-side images of the six pairs were also created with a red outline circle highlighting the difference. Two additional side-by-side image pairs were also generated. These were used when introducing the change blindness demonstrations to illustrate the type of changes that might occur in the sequentially presented displays. These two examples were chosen from pilot work and were rated the highest on relevance to driving and perceived difficulty in seeing the change. Two questionnaires (pre and post demonstration) were designed to elicit responses relating to participants’ confidence in their own observational abilities, and those of others whilst driving. The questions used a variety of scales. For the initial question, we wanted to include a complete list of scale descriptors (e.g., strongly agree) to start people thinking about their answers. Therefore, we chose a scale with five alternatives as this has been shown to be the most readily comprehendible to participants (Dawes, 2008). In this question we asked participants how difficult they thought spotting important visual changes would be by giving them five options: very difficult, difficult, neither difficult or easy, easy or very easy. The following questions used a 7-point Likert scale, as previous research has shown that this is the ideal number of alternatives for this type of question (e.g., Colman, Norris, & Preston, 1997; Ghiselli, 1955).^1^ For a full list of questions, please see Additional file 1. The demonstrations were presented on a 42-inch screen which was easily visible to all participants. The questionnaires were delivered in the form of multi-section paper booklets given to each participant.

#### Design and procedure

The demonstration was presented approximately halfway through the Driver Awareness Course run by Dorset Police (UK) Driver Education Unit and was delivered by the course instructors. In order to introduce and explain the study to the course instructors, the lead researcher made a site visit to go through the images that would be used and explain the purpose of the study. This also provided useful feedback from the course practitioners. In addition, an instruction sheet was provided to instructors which gave detailed information regarding obtaining informed consent from participants, when to deliver flashing imagery warnings and, importantly, outlining the procedure that the instructors should follow when delivering the change blindness demonstrations.

At a predetermined point in the Driver Awareness Course, instructors handed out the booklets to all participants; the study was then explained and the participants were asked whether they would like to volunteer to take part. Participants were informed that they would not be adversely affected in any way if they did not take part and that their answers would be analyzed anonymously. These two points were emphasized strongly to attempt to reduce biases caused by participants’ potential concerns that instructors might be made aware of their responses. Booklets were collected from those who did not wish to take part. Warnings stating that the demonstrations contained flashing imagery were given at various times throughout the procedure and participants were advised not to take part if they thought that they might be sensitive to this.

Instructors asked participants to open their booklets and fill in the pre-test questionnaire. Participants were shown two examples in the form of side-by-side images to demonstrate the types of changes they might expect in the change blindness task. The instructors then explained the change blindness task to the participants and that they should try to identify what was changing in each scene. After each demonstration, the two images from that demonstration were presented in a side-by-side format and the change was highlighted for the participants. After all six change blindness examples had been presented, participants completed the post-test questionnaire, followed by debriefing and continuation of the Driver Awareness Course. Participants were not able to see their responses to the pre-test questionnaire during this time.

### Results

**Fig. 2:**
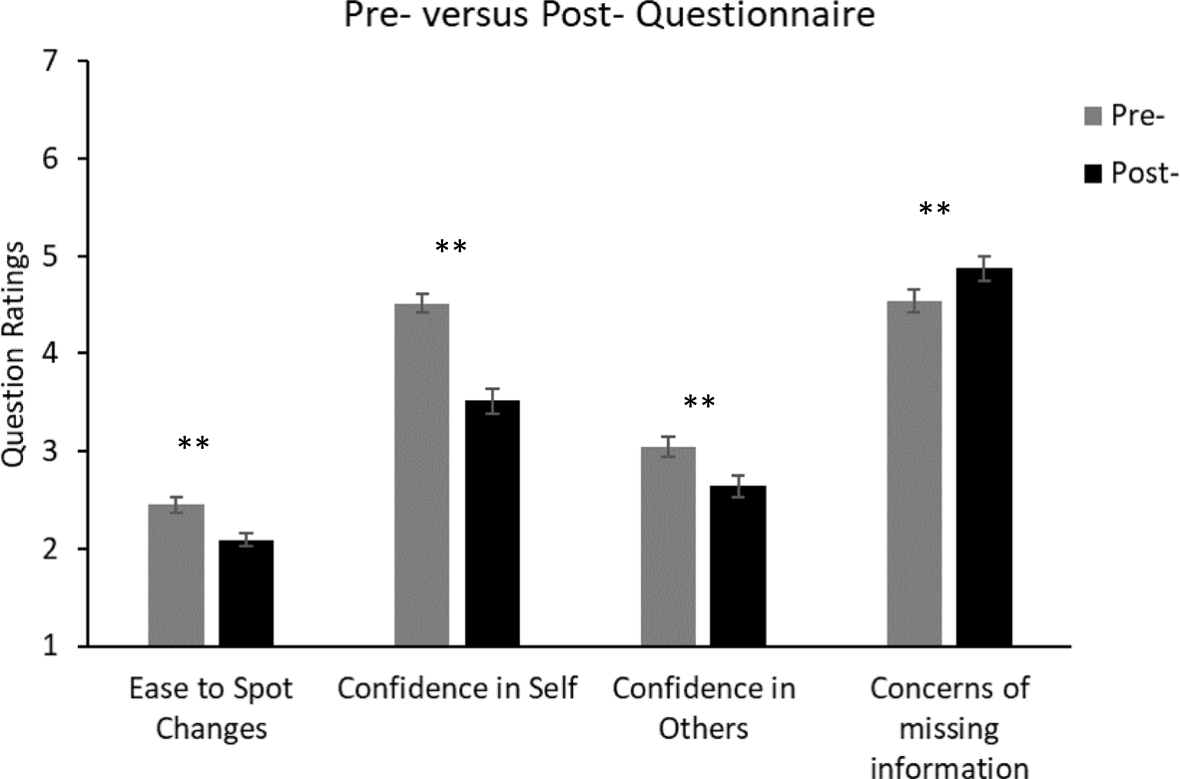
Average ratings from the pre-demonstration and post-demonstration questionnaires of Experiment 1. For the “ease to spot changes” question, the rating scale ranged from 1 (very difficult) to 5 (very easy). For the other questions, the rating scales ranged from 1 (not at all confident/concerned) to 7 (totally confident/concerned). Significant difference: **p < 0.01. Error bars represent the standard error. See Additional file 1 for the full questions

We first compared answers to questions that were present in both the pre-demonstration and post-demonstration questionnaires. We then considered responses to questions that were present in only the post-test questionnaire, followed by the open question responses. Figure 2 shows the pre-test and post-test questionnaire ratings for Experiment 1.

#### Comparisons between the pre-demonstration and post-demonstration questionnaires

The answers to questions pre and post demonstration were analyzed using mixed ANOVAs with the time point (pre or post demonstration) as the within-subjects factor, and age and gender as the between-subject factors. Gender comprised two categories, male (*N* = 96) and female (*N* = 61), and age was split into three categories that have been used previously (Shinar, Schechtman, & Compton, 2001) in driving-related research: 18–25 years (*N* = 20), 26–50 years (*N* = 79), 51 years and older (*N* = 51^2^).

##### Spotting important changes

A 2 (pre/post demonstration) × 2 (gender) × 3 (age) mixed ANOVA revealed that, having seen the demonstrations, participants reported that “Spotting important visual changes …” was more difficult than they had previously thought, *F*(1,137) = 12.29, mean squared error (MSE) = 0.418, *p =* 0.001, *η*_*p*_^2^ = 0.082 (with “very difficult” assigned the number 1 and “very easy” assigned the number 5). However, no other main effects or interactions were significant (all *F* ≤ 2.30, *p* ≥ 0.132).

##### Confidence in own and in others’ abilities

A 2 (time point: pre/post test) × 2 (you or others) × 2 (gender) × 3 (age) mixed ANOVA showed that ratings of confidence that “you/others see everything whilst driving” decreased between the pre-demonstration and post-demonstration questionnaires, *F*(1,141) = 65.69, MSE = 0.865, *p <* 0.001, *η*_*p*_^2^ = 0.318. In addition, participants gave significantly higher ratings of confidence in their own ability relative to their ratings of others, *F*(1,141) = 88.01, MSE = 1.74, *p <* 0.001, *η*_*p*_^2^ = 0.384. A significant time point × you or others interaction was also found, *F*(1,141) = 22.33, MSE = 0.446, *p <* 0.001, *η*_*p*_^2^ = 0.137. The demonstration produced a greater reduction in participants’ confidence in their own abilities (mean reduction = 1.04, standard error (SE) = 0.102) than in the confidence of others’ abilities (mean reduction = 0.376, SE = 0.092), *t*(148) = 5.905, *p* < 0.001, *d* = 0.48. No other main effects or interactions were significant (all *F* ≤ = 3.45, *p* ≥ 0.065).

##### Failing to see information

A 2 (pre/post test) × 2 (gender) × 3 (age) mixed ANOVA revealed that participants’ concern that they might “miss important visual information” increased significantly between the pre-demonstration and post-demonstration questionnaires, *F*(1,141) = 9.347, MSE = 0.770, *p =* 0.003, *η*_*p*_^2^ = 0.062. However, no other main effects or interactions were significant (all *F* ≤ 2.62, *p* ≥ 0.107).

At the end of the questionnaire, participants were asked to state whether they agreed or disagreed with three statements. Participants answered on a 5-point scale from strongly disagree, disagree, neither agree nor disagree, agree to strongly agree, and each response was given a numeric value from 1 to 5 respectively. On average, participants agreed; 114 out of 151 participants selected agree or strongly agree, *χ*^*2*^(4) = 118.172, *p* < 0.001 (mean = 3.9, SD = 0.9) that they were surprised by “how difficult it is to see/observe visual changes”. In response to the statement “Spotting important changes in a visual scene is easier than I expected it to be”, 99 out of 150 participants selected that they disagreed or strongly disagreed, *χ*^*2*^(4) = 72.533, *p* < 0.001, (mean = 2.3, SD = 1.1). Finally, 117 out of 151 participants stated that they agreed or strongly agreed that “I am now more aware of my visual limitations”, *χ*^2^(4) = 105.921, *p* < 0.001 (mean = 4.0, SD = 0.9).

Univariate ANOVAs were used to investigate these final questions further. Gender and age were included as fixed factors. No main effects or interactions were found for any of the final questions (all F ≤ 2.15, *p* ≥ 0.135).

#### Open question analysis

In the post-demonstration questionnaire, we asked two open-ended questions: “Did you find the demonstrations useful?” and “Do you think that the general public would benefit from viewing the demonstrations?” Thematic analysis (Boyatzis, 1998) was used to group and evaluate the responses given to these questions. Details of the identified themes can be found in Additional file 2.

#### Did you find the demonstration useful?

The majority of participants stated that they found the demonstration useful (130 out of 155, *χ*^*2*^(1) = 71.13, *p* < 0.001). All 25 people who answered “No” gave open responses which were grouped into two central themes: concerns relating to the purpose or general applicability of the change blindness demonstration; and issues with the design of the change blindness demonstration. For the 130 participants who reported finding the demonstration useful, 120 also gave open responses which were grouped into four central themes: that the demonstration raised awareness that it is important to maintain concentration whilst driving and continually be observant of your surroundings; that the change blindness task actually demonstrated how different people see the world; the perceived applications of the change blindness demonstration; and that the demonstration had made them question how confident they are in their ability to observe everything in the world around them.

#### Do you think that the general public would benefit from viewing the demonstrations?

Participants overwhelmingly stated that they believed that the general public would benefit from seeing the demonstration (128 out of 151 answered yes, *χ*^*2*^(1) = 73.01, *p* < 0.001). Of the 23 participants who responded “No”, 21 people gave open responses which were grouped into two themes: the aim of the demonstration was unclear; and the demonstration was not realistic enough or representative of real driving scenarios. Of the 128 participants who believed that the demonstration would be beneficial, 117 of them provided an open response which was grouped into three main themes: the demonstration would help to show the general public the importance of maintaining concentration on the roads and how difficult it can be to observe in detail a visual scene; how easy it is to miss important information and that people are overconfident in their ability to observe changes in the world around them; and the demonstration illustrated the differences between the participant and others’ ability to detect changes, and importantly that not everyone views a scene in the same way.

### Discussion

Experiment 1 found that showing participants a change blindness demonstration during a driving course had an impact on their self-rated observational skills. Participants reported that they found it to be more difficult to “spot important visual information” after they had viewed the demonstrations. Furthermore, participants’ confidence in their own and in others’ abilities decreased when asked whether “you/others see everything whilst driving”, and concern that they might “miss important visual information” increased after viewing the demonstration. The majority of participants also found the demonstration useful and indicated it would be beneficial to show it to the general public. Overall, the results support the hypothesis that showing people the change blindness demonstration had a positive effect on their self-knowledge about their observational patterns when thinking about driving.

However, as all participants completed both the pre and post test, it could be argued that their responses might be biased due to the “test–retest” design (e.g., due to perceived researcher expectations, social desirability effect, etc.). Furthermore, as the experiment was delivered by the police, participants may have let the perceived influence of authority affect their answers. In order to rule these factors out, in Experiment 2 we replicated the experiment, in a (non-police) laboratory environment, with participants recruited from the University of Warwick. We also included a condition in which participants only completed the questionnaire after the intervention (and not before).

Experiment 2 was also used to investigate whether any type of intervention would lead to a change in people’s driving perception. We believe that the change blindness demonstration should be particularly effective in making people reassess their visual abilities, due to the fact that you can show people that even large changes in their visual field can be easily missed. However, one could argue that other tasks would have the same effect. For example, as our study used driving stimuli, other tasks that involved the processing of driving-related stimuli might result in people thinking about their driving behavior. Second, the change blindness demonstration may have led participants to feel like they had “failed” at the task and this may have led to the reduced confidence in their reported abilities. To investigate these possibilities, we included two new conditions in Experiment 2: a visual search task; and a difficult question task. In a typical visual search task, participants are asked to search for a target item among competing distractor items (e.g., Duncan & Humphreys, 1989; Kunar & Humphreys, 2006; Treisman & Gelade, 1980; Wolfe, Birnkrant, Kunar, & Horowitz, 2005; Wolfe, Cave, & Franzel, 1989). In our visual search task, we asked participants to view the same driving stimuli as in Experiment 1; however, instead of a change blindness task, they searched the display for a target item (e.g., a road sign). In the difficult question task, participants were given six difficult driving theory questions that related to the UK Highway Code. The questions were modified to suggest that safe drivers should know the correct answer; however, in reality the questions were very difficult to answer. This was designed to generate a sense of “failure” in the task. Thus, if a sense of failure per se led people to being more cautious post intervention in Experiment 1, we should find a similar effect in this condition too.

## Experiment 2

### Participants

A total of 120 participants (58 female, 60 male, 2 declined to answer; age 18–37 years, mean = 21.1 years, SD = 4.4 years) from the University of Warwick staff and student population were recruited using an Online University Participant Panel whereby participants self-select to take part in studies. All participants held a driving license that allowed them to drive in the UK and received monetary compensation for their time. Participants were randomly assigned to one of the four conditions, resulting in 30 participants per condition. Ethical approval was granted from the University of Warwick’s Humanities and Social Sciences Research Ethics Committee.

### Materials and stimuli

The materials and stimuli were identical to those in Experiment 1 for the *replication*, *post-test only* and *visual search* conditions. However, the difficult question condition consisted of six UK driving theory questions that related to the Highway Code. The questions were selected from the most difficult theory test questions (https://highwaycodetest.co.uk/most-difficult-theory-test-questions/).

### Design and procedure

In the replication condition, the design and procedure were identical to those of Experiment 1. The post-test only condition was similar except that participants only completed the post-test questionnaire and did not complete the pre-test one. In the visual search condition, participants were shown the same stimuli as in replication and post-test only conditions but instead of detecting a change they were given a description of a target to search for (e.g., “Please click on the speed limit sign”). Participants then completed the post-test questionnaire. In the difficult question condition, participants were given six questions taken from the UK driving theory test. Under each question they were given four possible answers and asked to click on the one they believed to be correct. In the instructions for this task, participants were asked to imagine they were in a driving situation where they encountered a situation that, for safety, required an immediate and appropriate response. They were also informed that competent drivers should find this task easy. Error feedback was given to the participants after each question. Participants then completed the post-test questionnaire.

### Results

**Fig. 3:**
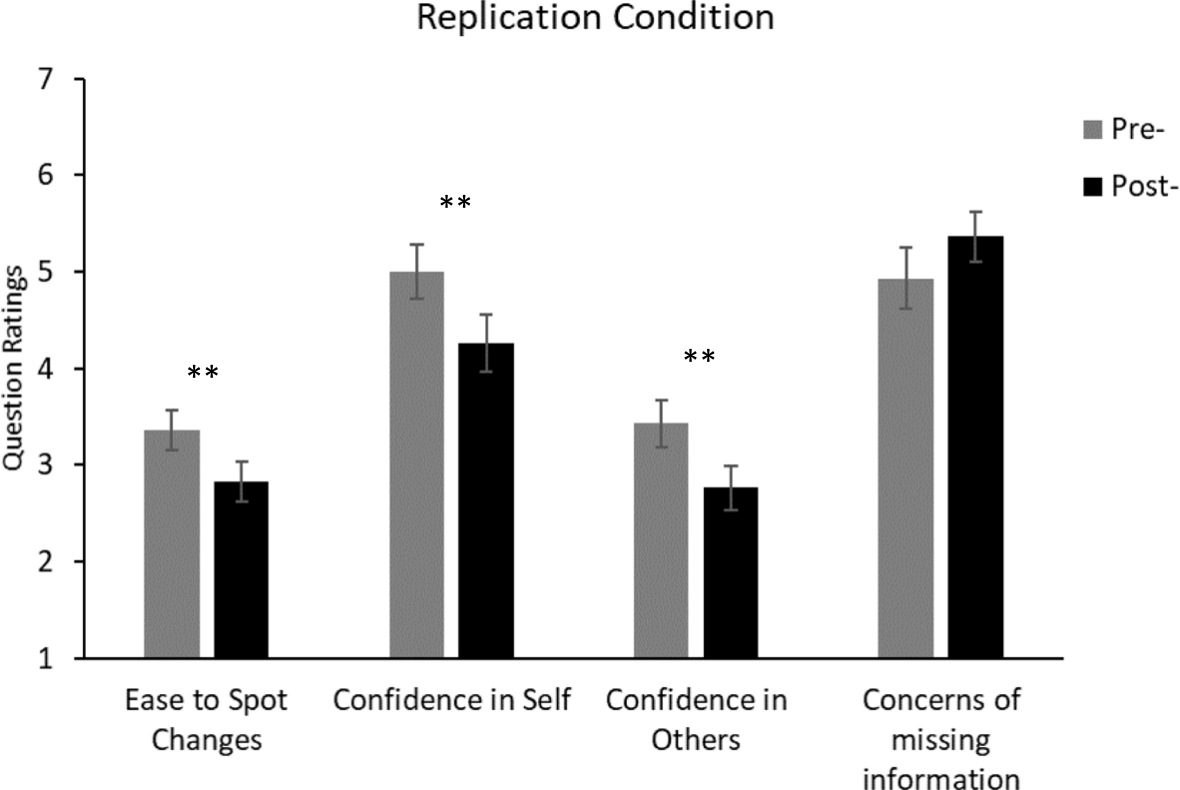
Average ratings from the pre-demonstration and post-demonstration questionnaires of the replication condition in Experiment 2. For the “ease to spot changes” question, the rating scale ranged from 1 (very difficult) to 5 (very easy). For the other questions, the rating scales ranged from 1 (not at all confident/concerned) to 7 (totally confident/concerned). Significant difference: **p < 0.01. Error bars represent the standard error. See Additional file 1 for the full questions

**Fig. 4:**
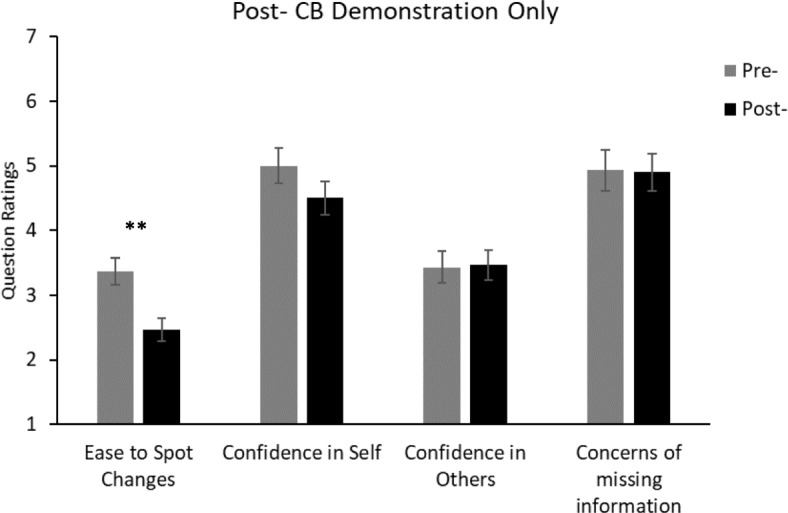
Average ratings from the post-demonstration data of the post-test only condition in Experiment 2. The pre-questionnaire data were taken from the replication condition for statistical comparison. For the “ease to spot changes” question, the rating scale ranged from 1 (very difficult) to 5 (very easy). For the other questions, the rating scales ranged from 1 (not at all confident/concerned) to 7 (totally confident/concerned). Significant difference: **p < 0.01. Error bars represent the standard error. See Additional file 1 for the full questions. CB change blindness

**Fig. 5:**
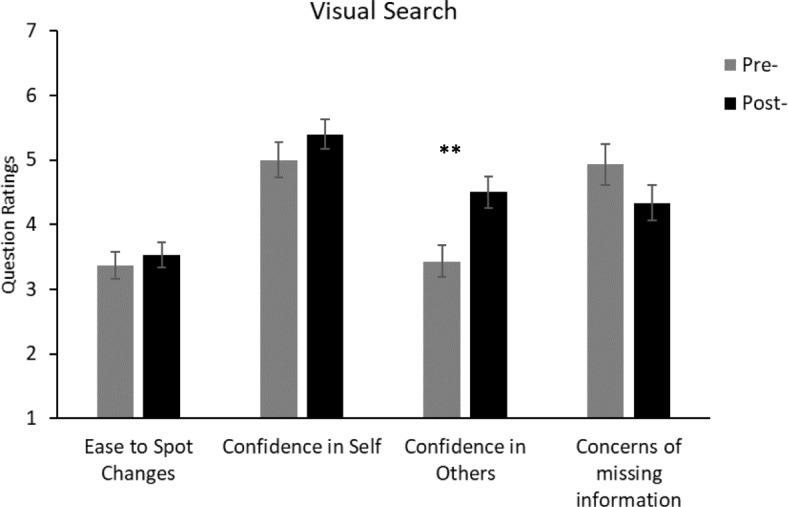
Average ratings from the post-demonstration data of the visual search condition in Experiment 2. The pre-questionnaire data were taken from the replication condition for statistical comparison. For the “ease to spot changes” question, the rating scale ranged from 1 (very difficult) to 5 (very easy). For the other questions, the rating scales ranged from 1 (not at all confident/concerned) to 7 (totally confident/concerned). Significant difference: **p < 0.01. Error bars represent the standard error. See Additional file 1 for the full questions

**Fig. 6:**
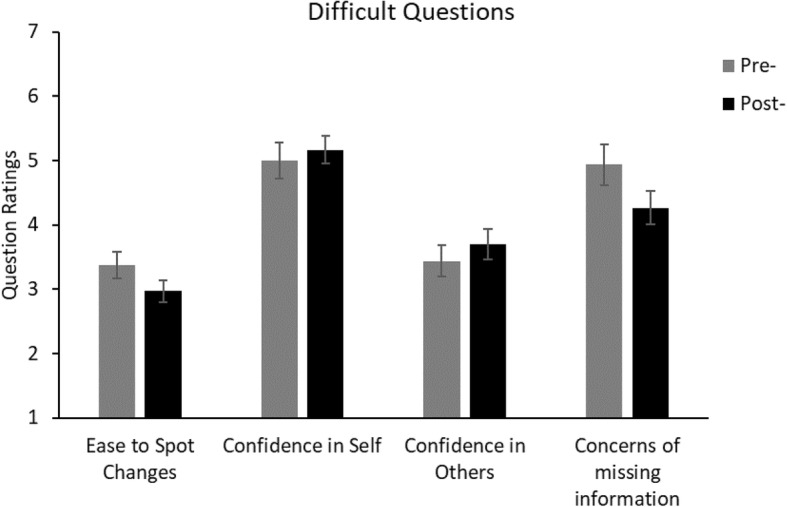
Average ratings from the post-demonstration data of the difficult questions condition in Experiment 2. The pre-questionnaire data were taken from the replication condition for statistical comparison. For the “ease to spot changes” question, the rating scale ranged from 1 (very difficult) to 5 (very easy). For the other questions, the rating scales ranged from 1 (not at all confident/concerned) to 7 (totally confident/concerned). Error bars represent the standard error. See Additional file 1 for the full questions

There are a number of comparisons that could be made. However, in the following we concentrate on only those that answer the questions of interest. Gender and age were removed from analyses as they were not found to have a significant effect in the initial study. Furthermore, as the majority of the participants were within the same age range, an analysis of age was inappropriate. Figures 3, 4, 5 and 6 show the questionnaire ratings from all conditions in Experiment 2.

#### Replication of Experiment 1

The replication condition produced essentially the same results as Experiment 1. Participants in the post-test questionnaire reported that “Spotting important visual changes …” was more difficult than they had previously thought in the pre-test questionnaire, *t*(29) = 2.90, *p* < 0.01, *d* = 0.473. A 2 (time point: pre/post test) × 2 (you or others) ANOVA showed that ratings of confidence that “you/others see everything whilst driving” decreased between the pre-demonstration and post-demonstration questionnaires, *F*(1, 29) = 19.55, MSE = 14.7, *p* < 0.01, *η*_*p*_^2^ = 0.403, and that participants gave significantly higher ratings of confidence in their own ability relative to their ratings of others, *F*(1, 29) = 35.29, MSE = 70.53, *p <* 0.01, *η*_*p*_^2^ = 0.549. The interaction between time point and you/others was not significant, *F* < 1. Furthermore, **r**esponses in the post-test questionnaire showed that there was a trend for participants to be more concerned that they might “miss important visual information” compared to their pre-test responses, *t*(29) = 1.99, *p* = 0.056, *d* = 0.283.

At the end of the questionnaire, participants were asked to state whether they agreed or disagreed with three statements. The majority of participants agreed that they were surprised by “how difficult it is to see/observe visual changes”; 25 out of 30 participants selected agree or strongly agree, *χ*^*2*^(4) = 38.0, *p* < 0.01; (mean = 3.9, SD = 0.9). In response to the statement “Spotting important changes in a visual scene is easier than I expected it to be”, 19 out of 30 participants selected that they disagreed or strongly disagreed, *χ*^*2*^(3) = 17.2, *p* < 0.01 (mean = 2.5, SD = 0.9). Finally, 23 out of 30 participants stated that they agreed or strongly agreed that “I am now more aware of my visual limitations”, *χ*^2^(4) = 20.7, *p* < 0.01 (mean = 4.0, SD = 1.0). The majority of participants also stated that they found the demonstration useful (27 out of 30, *χ*^*2*^(1) = 19.2, *p* < 0.01) and believed that the general public would benefit from seeing the demonstration (26 out of 30, *χ*^*2*^(1) = 16.1, *p* < 0.01).

#### Comparison of pre-test and post-test questionnaires between the replication and the post-test only conditions

Responses from the post-test only condition were compared with the pre-test responses from the replication condition. Participants rated that “Spotting important visual changes …” was more difficult in the post-test questionnaire than in the pre-test questionnaire, *t*(58) = 3.31, *p* < 0.01, *d* = 0.867. However, there was no difference in responses for questions relating to confidence that “you/others see everything whilst driving” or that participants might “miss important visual information” in the pre-test and post-test questionnaires (all *t* < 1.4, *p* > 0.19).

#### Does visual search using driving-related images lead to a change in response?

Responses from the post-test questionnaire of the visual search condition were compared with the pre-test responses from the replication condition. Participants rated that they thought other people were more confident “that they saw everything whilst they are driving” in the post-test questionnaire than in the pre-test questionnaire, *t*(58) = 3.13, *p* < 0.01, *d* = 0.823. However, there was no difference in responses for any of the other pre-test or post-test questions (all *t* < 1.5, *p* > 0.15).

#### Does a sense of “failing” lead to a change in response?

There were no significant response differences between the post-test responses of the difficult questions condition and the pre-test responses of the replication condition (all *t* < 1.7, *p* > 0.1).

### Discussion

The results of the replication condition were essentially the same as those of Experiment 1. That is, having seen the change blindness demonstrations, compared to their pre-test responses the participants: reported that spotting important visual information was more difficult than they had previously thought; reported confidence that “you/others see everything whilst driving” decreased; and were marginally more concerned with missing important visual information compared to their pre-test responses. Participants also found the demonstration to be useful and believed that the general public would benefit from seeing it.

Comparing the pre-test responses in the replication condition with the post-test responses in the post-test condition also showed that participants considered that spotting important visual information was more difficult following the change blindness demonstrations. However, there was no difference in response evaluations in relation to confidence in self/others or being concerned about missing important information. Examining participants’ responses after the other “interventions” (visual search and driving questions), we see little evidence of a change in self-reported observational ability. The one exception being that participants thought other people may be more confident in seeing everything whilst they are driving after viewing the visual search task. We discuss this further in the General discussion.

## General discussion

The main aim of the current study was to develop and assess the feasibility and effectiveness of presenting a change blindness demonstration within a driver education course. We predicted that demonstrating to participants that their visual system is not infallible might reduce unfounded confidence in their observational abilities. A necessary first step for our analysis was establishing whether one of the key learning points from the change blindness intervention had been achieved. This was whether participants had been made aware of how difficult it can be to detect important changes in our immediate environment. Our results suggest that this message was delivered successfully. Participants, on average, stated that it was more difficult to “spot important visual information” after they had viewed the demonstrations. This occurred when the questionnaires were administered in a within-participants design (Experiments 1 and 2) and in a between-participants design (Experiment 2), in which there was no opportunity for a pre-test/post-test response bias to occur. Furthermore, this change was observed only following the change blindness intervention and not after the other “pseudo-interventions” (visual search or difficult questions).

In terms of whether participants think they or others “see everything whilst driving” or whether they might “miss important visual information”, we find the results to be mixed. When the questionnaires were given in a within-participant design following the change blindness intervention, the results indicated that participants reported a decrease in self or others seeing everything while driving and an increase in whether they might miss important information. Again, this only happened following the change blindness intervention and not after the “pseudo-interventions”. However, given that this was not replicated when examining the between-participants comparison, it is best to treat this particular result with some caution. Regardless of this, however, there is strong evidence that, overall, showing the change blindness intervention made participants aware of the difficulty in spotting important visual information.

Simple knowledge of the negative effects or risks of performing a particular behavior can play an important role in affecting how likely someone is to perform the said behavior. For example, a key factor found to motivate people to give up smoking was the bringing of smoking-related risks into the public domain via clear and powerful package labeling (Hammond, McDonald, Fong, Brown, & Cameron, 2004). However, it was important that the demonstration also impacted on participants’ attitudes, while increasing their knowledge base. Attitudes play an important role in behavioral change and feature prominently in many models of behavior (Ajzen, 1991; Michie et al., 2011). Our analysis demonstrated that participants stated that spotting important changes was harder after the experiment. Moreover, a qualitative analysis provided a wealth of examples of participants stating that they were surprised at how difficult it is to see changes like those demonstrated and that they are not as observant as they had previously thought.

This is a very encouraging finding and answers the main question posed by this study—whether attitudes toward observation and concentration, namely overconfidence in the said abilities, can be attenuated by exposure to change blindness demonstrations. In addition, although the impact of this study on participants’ real-world driving behavior was not investigated, the theme identified from our qualitative data regarding the need for greater observation and concentration while driving implies that participants may be critically evaluating the need to change their behavior as a result of the adjustment in confidence bought about by the change blindness demonstration.

There are many examples of studies which have shown that attitudes are linked to intentions to perform certain behaviors—for example, speeding (De Pelsmacker & Janssens, 2007) or texting while driving (Nemme & White, 2010). So, it is not unreasonable to predict behavioral change as a result of the attitudinal/confidence changes brought about by the demonstration. However, this was not explicitly tested for, as the main focus of the demonstration was lowering overconfidence, which has been suggested to be a contributing factor to traffic accidents (Deery, 2000; Harré, Foster, & O’Neill, 2005).

Our findings suggest that participants overestimated their ability to detect changes in a visual scene. This meshes with findings from previous change blindness studies (e.g., Levin et al., 2000) and the driving skill literature (Svenson, 1981). Stevenson, Palamara, Morrison, and Ryan (2001) found that drivers who had medium to high ratings of confidence-adventurousness were around twice as likely to have a vehicular collision as those with lower ratings. In fact, this “overconfidence” has been suggested as a major factor in road safety and driving-related decisions by a variety of sources (Deery, 2000; Harré et al., 2005; Katila et al., 1996; Sandroni & Squintani, 2004; see also, Vrolix, 2006, for related work), and therefore it is very encouraging that a change blindness intervention was able to reduce overconfidence in a key driving-related ability, at least in the short term. However, of course, future research will need to determine the robustness of this change.

### Factors that might influence the effectiveness of change blindness demonstrations

In an attempt to be as effective as possible, the change blindness images were all of driving-related situations (McCarley et al., 2004). They were also designed to cover a range of perceived difficulties so that some demonstrations contained “obvious changes” that participants would expect to notice easily but were, in reality, difficult to detect. Overall, participants were surprised by how challenging the changes were to see and stated that it was more difficult to observe the changes than they had originally thought. One might argue whether it was the driving-related stimuli or the surprising difficulty of the change blindness intervention that shifted people’s self-ratings. However, given that the same results did not occur in the visual search task (which used the identical driving stimuli as in the change blindness condition) or in the difficult questions task, we believe these factors were not responsible for the results. In any case, it would seem sensible to construct change blindness tasks using content that relates as closely as possible to the relevant context/domain of education to be targeted so that participants can more easily see the applicability of the demonstration.

A flaw in many interventions designed to affect behavior is that they can be avoided or their message denied by the individual who is being targeted. Ruiter, Abraham, and Kok (2001) reviewed the literature on interventions which induce fear in their target audience in an attempt to influence future behavior. Among the potential problems with fear-inducing campaigns is that participants may deliberately avoid the campaign as a defense mechanism to control their own fear level. Therefore, the message will not be delivered successfully. Other studies of mass-media road interventions have demonstrated that they may not be effective at reaching certain sectors of the population such as people with lower degrees of education who are less likely to pay attention to a campaign (Weenig & Midden, 1997; see Hoekstra & Wegman, 2011, for a review of road safety campaigns). In addition, Harré et al. (2005) found that when their participants viewed short films designed to demonstrate the dangers of drink driving, they reported inflated opinions of their own driving skill. The authors suggested that this may be due to the fact that participant’s may judge others as having poor driving skills and therefore consider their own skill level to be higher. The change blindness demonstration implemented in the driver education course may benefit from the fact that it was delivered in a group setting, which allowed discussion and encouraged engagement with the material, while at the same time engaging participants and simultaneously demonstrating to each of them flaws in their visual awareness. By demonstrating the flaw rather than simply describing a behavior and presenting examples of how others are affected by it, the participants could not as easily dismiss it as something that was not relevant to them and assume that the message was meant for other people. In fact, personalizing the message has been suggested as a worthy pursuit for driver education campaigns (Hoekstra & Wegman, 2011). Furthermore, because change detection performance does not appear to improve with practice (Rensink et al., 1997), the limits of visual processing could be experienced repeatedly by each group member, further reinforcing the message.

### Framing of the demonstration

As noted earlier, some participants reported that the procedure was not representative of real-world driving scenarios and was unrealistic. We note that the demonstrations were not solely designed to be completely representative of changes that may occur whist driving. Rather, they were designed to demonstrate, more generally, how easy it is for even relatively large changes to occur and yet not be perceived within a driving-related context. This appears to have been well received by the majority of participants and was clearly identified as a theme in the responses by those participants who reported that they found the demonstration useful. However, for a minority this point appears to have been missed, perhaps as a result of variation in the presentation style of individual instructors. Although the instructors were briefed on the procedure, it is to be expected that there would be some variation in presentation style and emphasis. Although effective for the majority of participants in this study, it seems that ensuring participants understand the general point of the demonstration will maximize its benefits. One way to achieve this might be to make participants aware of research findings, such as that holding a mobile phone conversation disrupts people’s ability to detect changes in traffic scenes (McCarley et al., 2004) and interferes with how you pay attention to the world (regardless of task or conversation difficulty; Kunar, Carter, Cohen, & Horowitz, 2008; Kunar, Cole, Cox, & Ocampo, 2018; Strayer & Johnston, 2001). This may indicate to participants why they should think carefully about how observant they are whilst driving. At the same time, the results would demonstrate a clear link between change blindness task performance and driving scenarios.

### Demographic differences in responses

Contrary to what could have been predicted from previous literature (Barber & Odean, 2001; Bengtsson et al., 2005; Lundeberg et al., 1994; Niederle & Vesterlund, 2007), including specifically studies which have found gender differences in how driving risk is estimated (Finn & Bragg, 1986; Rosenbloom et al., 2008), we did not find a significant difference between male and female participants’ responses.

Given that previous studies have indicated differences in overconfidence between different age groups (e.g., Menkhoff et al., 2013; Pliske & Mutter, 1996) in driving-related judgments (Finn & Bragg, 1986; Rosenbloom et al., 2008), we also examined the effect of age on confidence and the influence of the demonstration. However, we found no evidence for an effect of age on confidence judgments and neither did age interact with the reported effectiveness of the demonstration. There are a number of reasons why this might be. First, it may be because the majority of participants in the upper age category were in their 50s and 60s. A difference might have emerged if our sample had contained a greater proportion of older adults (e.g., those aged over 70 years). Second, it may be that as all participants had been offered the course as a result of committing a driving-related infraction, this experience might have acted to level any differences in confidence across the age groups. Finally, it might be because change blindness is a particularly effective tool for this type of demonstration as the majority of people typically *believe* they would be able to detect the changes, yet fail to do so in practice (Levin et al., 2000). Driver Awareness Courses are populated by a diverse set of attendees, both in terms of age and gender. As such, it is critically important that an intervention designed for use within a Driver Awareness Course (such as the change blindness task we present in this article) is not biased to affect one subset of drivers over another. It is encouraging, then, that our results indicate that the change blindness intervention which we present here is likely to be effective across a large and diverse range of participants, irrespective of age and gender.

### The reliability of drivers’ self-reported data

It is well known that self-report data can be subject to biases such as participants being untruthful in their responses and answering in such a way as to conform to social expectations (Nederhof, 1985). However, self-report questionnaires are nevertheless a useful tool for the assessment of interventions and participant attitude change that can be very difficult to measure effectively in other ways. In addition, there is a precedent for the validity of self-report data in the driving domain. Lajunen and Summala (2003) asked two groups of people—applicants to a driver instructor training course and students on the course—to fill in the Driver Behavior Questionnaire (DBQ; Reason, Manstead, Stradling, Baxter, & Campbell, 1990) and a scale designed to measure the extent to which participants were trying to give socially desirable answers. The applicants completed the questionnaires in public and the students completed them in private. There were few differences between the two groups but those who completed the questionnaires in public reported negative behaviors less frequently. Overall, the DBQ responses showed only a relatively small bias toward socially desirable responding.

This is encouraging for the current study as although participants completed the questionnaire in a group setting, they were assured that their answers would be anonymous. This inspires confidence that the answers given were not simply a result of participants answering questions in what they perceived to be the socially desirable way. Evidence from the current study which supports this assumption is that not all answers followed what might have been perceived as a correct or desirable response.

### Impact on driving and driving education

The results indicate that showing people a change blindness task led to them becoming more aware of their visual limitations. The hope is that by becoming self-aware of their own observational fallibility, participants will pay more attention to the road when they are driving. Although it would have been good to obtain a direct measure of driving performance and behavior change following the intervention in the real world, unfortunately this was not possible given the scope of the study. Hence, it remains possible that although effective in the short term as measured by self-reports, the intervention has little impact on real-world driving behavior. Accordingly, an important goal of future research will be to study the effects of change blindness interventions on actual driving performance and driving reoffending rates. Nevertheless, as the goal of the Driving Education Course was to explore and provide advice to people on their driving skills and behavior, the change blindness demonstration offered a low-cost opportunity for participants to interactively reflect on their observational abilities—an opportunity which was deemed beneficial by the majority of participants. In short, the main aim of our study was to create and evaluate an intervention task which would: be short in duration so that it could be incorporated into an existing course; be presented to a relatively large number of people simultaneously; be relatively resource light; illustrate a clear limitation in people’s attentional/perceptual abilities; and prompt people to reassess their observational skills. In these respects, our study has shown that change blindness interventions can be particularly effective for achieving these types of goals.

## Conclusion

This study examined the feasibility and potential benefits of implementing a change blindness demonstration in a national driver safety course. The overall findings showed that such a task was effective in highlighting participants’ overconfidence in their own observation abilities to notice important visual information. The majority of participants reported that the demonstration was useful and that it would be valuable to present to other road users and the general public. Examining the longer term effects beyond the local context of the Driver Awareness Course in comparison to other parts of such courses was beyond the scope of the current study and will be a goal for future research. Nonetheless, at this stage we are confident that the change blindness task was effective in raising the awareness of observational limits and changing driver’s attitudes, and has the potential to cause positive behavioral change.

## Additional files

Additional file 1: Questionnaire used in this study. Part 1 is the pre-demonstration questionnaire and part 2 is the post-demonstration questionnaire (DOCX 23 kb)

Additional file 2: Example responses to the open-answer questions from Experiment 1. Data were sorted into themes by the experimenter and an independent coder who was blind to the purpose and design of the experiment. The two coders worked independently at first and then discussed the results of their individual thematic categories (DOCX 17 kb)

## Abbreviations

ANOVA: Analysis of variance
COM-B: Capability Opportunity Motivation-Behavior

## Footnotes

1 Please note that both 5-point and 7-point scales are considered optimal scales for Likert questionnaires with any increase in the number of categories above seven considered ineffective (Colman et al., 1997).

2 For the older age category, the distribution of ages was made up of 28 participants aged 51–60 years, 19 participants aged 61–70 years and four participants aged 70 years or older.
