# Supplementary material for: The hazards of perception: evaluating a change blindness demonstration within a real-world driver education course
Source: Cogn Res Princ Implic. 2019 May 21;4:15. doi: 10.1186/s41235-019-0165-4 (PMC6529486; doi:10.1186/s41235-019-0165-4)
Supplement: Supplementary file 1 — Questionnaire used in this study. Part 1 is the pre-demonstration questionnaire and part 2 is the post-demonstration questionnaire (DOCX 23 kb) [file 41235_2019_165_MOESM1_ESM.docx]

**Appendix 1**

Below is a copy of the Questionnaire that was used in this study. Part 1 is the Pre-demonstration questionnaire and part 2 is the Post-demonstration questionnaire.

**Questionnaire: Part 1**

Please circle your Gender. Male Female Other ____________

Please indicate your age in years ___________

Please select the phrase which you believe best fits in the gap in the sentence below.

Spotting important visual changes, such as a child running out from behind a parked car, is ___________.

| Very Difficult | Difficult | Neither difficult nor easy | Easy | Very Easy |
| --- | --- | --- | --- | --- |

How confident are you that you see everything whilst you are driving? (please circle a number on the scale below)

| Not confident at all |  |  |  |  |  | Totally confident |
| --- | --- | --- | --- | --- | --- | --- |
| 1 | 2 | 3 | 4 | 5 | 6 | 7 |

How confident are you that other people see everything whilst they are driving? (please circle a number on the scale below)

| Not confident at all |  |  |  |  |  | Totally confident |
| --- | --- | --- | --- | --- | --- | --- |
| 1 | 2 | 3 | 4 | 5 | 6 | 7 |

Are you concerned that you might miss important visual information? (please circle a number on the scale below)

| Not concerned at all |  |  |  |  |  | Extremely concerned |
| --- | --- | --- | --- | --- | --- | --- |
| 1 | 2 | 3 | 4 | 5 | 6 | 7 |

**Please stop here and wait for further instructions.**

**Questionnaire: Part 2**

**Now that you have seen the demonstrations please answer the questions below.**

Did you find the demonstrations useful?

Yes/ No

In the box below please tell us why you have answered either Yes or No.

Do you think that the general public would benefit from viewing the demonstrations?

Yes/ No

In the box below please tell us why you have answered either Yes or No.

Please select the phrase which you believe best fits in the gap in the sentence below.

Spotting important visual changes, such as a child running out from behind a parked car, is ___________.

| Very Difficult | Difficult | Neither difficult nor easy | Easy | Very Easy |
| --- | --- | --- | --- | --- |

How confident are you that you see everything whilst you are driving? (please circle a number on the scale below)

| Not confident at all |  |  |  |  |  | Totally confident |
| --- | --- | --- | --- | --- | --- | --- |
| 1 | 2 | 3 | 4 | 5 | 6 | 7 |

How confident are you that other people see everything whilst they are driving? (please circle a number on the scale below)

| Not confident at all |  |  |  |  |  | Totally confident |
| --- | --- | --- | --- | --- | --- | --- |
| 1 | 2 | 3 | 4 | 5 | 6 | 7 |

Are you concerned that you might miss important visual information? (please circle a number on the scale below)

| Not concerned at all |  |  |  |  |  | Extremely concerned |
| --- | --- | --- | --- | --- | --- | --- |
| 1 | 2 | 3 | 4 | 5 | 6 | 7 |

Now that you have seen the demonstrations please indicate in the table below how much you agree with each statement.

| **Having seen the demonstrations….** | **Strongly Disagree** | **Disagree** | **Neither Agree nor Disagree** | **Agree** | **Strongly Agree** |
| --- | --- | --- | --- | --- | --- |
| I am surprised by how difficult it is to see/observe visual changes. | **1** | **2** | **3** | **4** | **5** |
| Spotting important changes in a visual scene is easier than I expected it to be. | **1** | **2** | **3** | **4** | **5** |
| I am now more aware of my visual limitations. | **1** | **2** | **3** | **4** | **5** |
