# Supplementary material for: The hazards of perception: evaluating a change blindness demonstration within a real-world driver education course
Source: Cogn Res Princ Implic. 2019 May 21;4:15. doi: 10.1186/s41235-019-0165-4 (PMC6529486; doi:10.1186/s41235-019-0165-4)
Supplement: Supplementary file 2 — Example responses to the open-answer questions from Experiment 1. Data were sorted into themes by the experimenter and an independent coder who was blind to the purpose and design of the experiment. The two coders worked independently at first and then discussed the results of their individual thematic categories (DOCX 17 kb) [file 41235_2019_165_MOESM2_ESM.docx]

# Appendix 2

Example responses to the open answer questions from Experiment 1. The data were sorted into themes by the experimenter and an independent coder who was blind to the purpose and design of the experiment. The two coders worked independently at first and then discussed the results of their individual thematic categories.

**Did you find the demonstration useful?**

“No” - 25 out of 155 participants

| **Themes** | **Specific Comments** |
| --- | --- |
| Concerns relating to the purpose or general applicability of the change blindness demonstration | *“I didn’t think it resembled real life. I don’t look for observations like those when I am driving or out.”*  -18, Female |
|  | *“I don’t think the test is a fair reflection of how well people concentrate and observe at the wheel”*  -34, Male |
| Issues with the design of the change blindness demonstration. | *“A lot of the time the flashes were too quick and wouldn’t actually put you in a genuine situation”*  -24, Female |
|  | *“I didn’t find it a very realistic model of what happens on the road. A film or moving image where certain elements are suddenly removed would be better.”*  -37, Female |

“Yes” - 130 out of 155 participants

| **Themes** | **Specific Comments** |
| --- | --- |
| The demonstration raised awareness that it is important to maintain concentration whilst driving and continually be observant of your surroundings. | *“It teaches you how hard you have to concentrate if you are going to see the whole picture”* -34, Male |
|  | *“Yes- because it makes you more aware. When most of the time you do not concentrate sufficiently.”* -57, Female |
|  | *“Made me realise I need to be more observant. It’s easy not to see things that can be quite important.”* -30, Female |
|  | *“It has made me realise that I don’t see everything in my fields of vision with a single glance so I do need to spend more time looking.”* -30, Female |
| The change blindness task actually demonstrated how different people see the world. | *“It’s interesting to see just how different people perceive different situations”* -26, Male |
| Perceived applications of the change blindness demonstration. | *“Alerts you to potential dangers which will affect road safety”* – 50, Male |
|  | *I answered yes because it made me think more about the things around me, even though I considered I was road aware.”*  -52, Female |
| Confidence. | *“Made me realise how over confident I am at noticing things.”* -34, Female |

### Do you think that the general public would benefit from viewing the demonstrations?

“No” - 23 out of 151 participants

| **Themes** | **Specific Comments** |
| --- | --- |
| The aim of the demonstration was unclear. | “*Needs to be observed with a specific objective”* – 68, Male |
| The demonstration was not realistic enough, or representative of real driving scenarios. | *“Is not set in real time and made in same situation and condition as if you were driving ie. House disappearing.”* -27, Male |

“Yes” - 128 out of 151 participants

| **Themes** | **Specific Comments** |
| --- | --- |
| The demonstration would help to show the general public the importance of maintaining concentration on the roads and how difficult it can be to observe in detail a visual scene. | *“The exercise enables one to think about the fact that on first sight you are not gathering all the available or important information. It makes it clear that you need more time than you imagine to assess road risk.”*  -42, Male |
| The exercise demonstrated how easy it is to miss important information and that people in general are over confident in their ability to observe changes in the world around them. | *“To many people believe and think they are observant. To much confidence.”*  – 46, Male |
|  | *“In general it may be a perception that we believe we notice everything but reality is we don’t and this exercise proved it.”*  -56, Male |
| The demonstration was effective at illustrating the differences between the participant and others’ ability to detect changes and importantly that not everyone views a scene in the same way. | *“Raises awareness of lack of observational skills in self + other road users.”*  – 42, Female |
|  | *“Show people how other people see things.”* – 41, Male |
